# Modified Needleman–Wunsch algorithm for clinical pathway clustering

**DOI:** 10.1016/j.jbi.2020.103668

**Published:** 2021-03

**Authors:** Emma Aspland, Paul R. Harper, Daniel Gartner, Philip Webb, Peter Barrett-Lee

**Affiliations:** a School of Mathematics, Cardiff University, Cardiff, United Kingdom; b Velindre Cancer Centre, Cardiff, United Kingdom

**Keywords:** Clinical pathways, Data mining, Lung cancer

## Abstract

Clinical pathways are used to guide clinicians to provide a standardised delivery of care. Because of their standardisation, the aim of clinical pathways is to reduce variation in both care process and patient outcomes. When learning clinical pathways from data through data mining, it is common practice to represent each patient pathway as a string corresponding to their movements through activities. Clustering techniques are popular methods for pathway mining, and therefore this paper focuses on distance metrics applied to string data for k-medoids clustering. The two main aims are to firstly, develop a technique that seamlessly integrates expert information with data and secondly, to develop a string distance metric for the purpose of process data. The overall goal was to allow for more meaningful clustering results to be found by adding context into the string similarity calculation. Eight common distance metrics and their applicability are discussed. These distance metrics prove to give an arbitrary distance, without consideration for context, and each produce different results. As a result, this paper describes the development of a new distance metric, the modified Needleman–Wunsch algorithm, that allows for expert interaction with the calculation by assigning groupings and rankings to activities, which provide context to the strings. This algorithm has been developed in partnership with UK’s National Health Service (NHS) with the focus on a lung cancer pathway, however the handling of the data and algorithm allows for application to any disease type. This method is contained within Sim.Pro.Flow, a publicly available decision support tool.

## Highlights

• Complex clinical pathways may require variation reduction to aid understanding.
• Patient pathway strings enable k-medoids clustering using string distance metrics.
• Eight common distance metrics discussed ultimately lack consideration for context.
• Produces a modified Needleman–Wunsch algorithm combining data and expert knowledge.
• Case study applies the method to lung cancer.

## Graphical abstract

**Figure:**
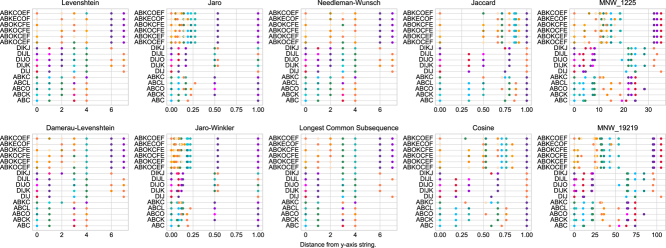

## 1 Introduction

Lung cancer is in the top ten causes of death, the most common cause of cancer death in men, and second most common in women, worldwide [1]. Cancer mortality can be reduced with early treatment and detection. As a consequence, the goal of many organisations that provide cancer services, is to reduce the time to diagnose and treat cancer.

In the age of digital health, the organisation of health information into interactive clusters and other novel methods for stratifying health data will complement existing approaches and potentially lead to improvements in health care [2]. As health information technology (IT), such as electronic health records (EHRs), gain widespread adoption and use in healthcare industry, thereby accumulating vast amounts of real-time patient care data, there is tremendous opportunity to develop data-driven models, methods and tools to facilitate review of practice workflows and improve evidence based care delivery by learning practice-based pathways of care [3], [4], henceforth denoted as clinical pathways.

When considering clinical pathway modelling, a primary question is often to consider what is the pathway. A recent review of the current literature [5] highlighted that there are many data mining and machine learning methods available for answering such questions. However, it was clear that most of these techniques only consider the pathways discoverable from data, and do not consider the wealth of information available from the experts that interact with the pathway day to day. The benefit of consulting with experts is that they may be able to explain some obscure or outlier information that can be picked up within the data. It is speculated that the lack of interaction between using both data and expert knowledge is due to the time consuming nature of such a process.

Clustering techniques were highlighted in the literature [5] as the most popular method for pathway discovery. Similarly, this paper focuses on distance measures applied to string data for the purpose of k-medoids clustering [6]. This method was chosen as firstly the data used is similar to that of Vogt et al. [7], and secondly using an existing pathway as the centroid reinforces the medical experts confidence in the pathway chosen as being realistic. Clustering methods do not hold the same limitation in regards to restricting that each activity can only be performed once that other methods have, making it more versatile and applicable.

This paper discusses the development of a new distance metric, modified from the Needleman–Wunsch algorithm, to allow for consideration of both data and medical expert information, for the use with clustering. Eight other popular distance metrics are discussed and used as reference for benchmarking the performance of the modified metric. The main dataset contains 2350 non-small cell lung cancer referrals provided by Velindre Cancer Centre (VCC), a cancer centre in the UK’s National Health Service (NHS).

The content is structured as follows: Section 2 contains a discussion of previous research, Section 3 gives a description of the problem, Section 4 discusses some current metrics and their properties, Section 5 details the development of the new algorithm, Section 6 applies the method to case studies. The paper closes with a conclusion and recommendations for further work.

## 2 Previous research

Aspland, Gartner and Harper [5] conducted an in-depth literature review on clinical pathway modelling which provides a taxonomy of problems related to clinical pathways and explores the intersection between methods drawn from Information Systems, Operational Research and Industrial Engineering. There were 82 papers in the review [5] which stated using data mining or machine learning, for mapping, modelling or improving the clinical pathway. Table 1 further categorises these papers into specific method areas.

It can be seen that clustering was the most popular method. On closer inspection there are multiple methods of clustering used, for example, Funkner et al. [10] use K-means, Vogt et al. use K-medoids [7] and Zhang et al. use hierarchical [3]. Furthermore, the differences go deeper when considering the distance measures used during clustering, as Funkner et al. [10] uses Levenshtein distance, Syed and Dias [43] modify the Needleman–Wunsch Algorithm, whereas Vogt et al. [7] and Zhang et al. [3] use Longest Common Subsequence (LCS).

**Table 1:** Publications categorised as data mining or machine learning method.

Method	
*Clustering*	[3], [7], [8], [9], [10], [11], [12], [13], [14], [15], [16], [17], [18]
*Categorised*	[19], [20], [21], [22], [23]
*Classified*	[24], [25]

*Topic modelling*	[26], [27]
*Probabilistic*	[20], [28], [29]
*Latent dirichlet allocation*	[25], [30], [31], [32], [33], [34], [35], [36]

*Pattern mining*	[14], [21], [37]
*Sequential pattern mining*	[17], [38], [39], [40], [41], [42], [43], [44], [45]
*Temporal pattern mining*	[39], [46], [47]

*Process mining*	[13], [14], [15], [35], [48], [49], [50], [51], [52], [53], [54], [55], [56]

*Bayesian*	[22], [57], [58], [59], [60]
*Markov*	[3], [61], [62], [63], [64]

*Heuristics*	[10], [65], [66], [67], [68]

*Semantic web rule language*	[69], [70]

*Artefact*	[60], [71], [72], [73], [74]

*Business Process Model and Notation (BPMN)*	[75], [76], [77]

*Other*	[23], [78], [79], [80], [81], [82], [83], [84], [85], [86], [87]

Aspland, Garter and Harper [5] also highlighted that there are two common ways of obtaining the pathway: either data-driven or through collaboration with experts who regularly interact with the pathway. Data-driven pathway discovery was most popular, containing 90 papers, compared to 13 papers that considered collaboration only.

Aspland, Gartner and Harper [5] state that there are 14 papers that considered information from both of these sources [52], [53], [71], [88], [89], [90], [91], [92], [93], [94], [95], [96], [97], [98]. All of these papers consider data alongside expert opinion, interviews or literature, and do so in a way that they enhance or fill in for missing information.

None of the papers integrate the two sets of information in a simple and direct manner. Furthermore, considering just one of these methods leaves a wealth of knowledge that is not considered.

## 3 Problem description

**Fig. A.15:**
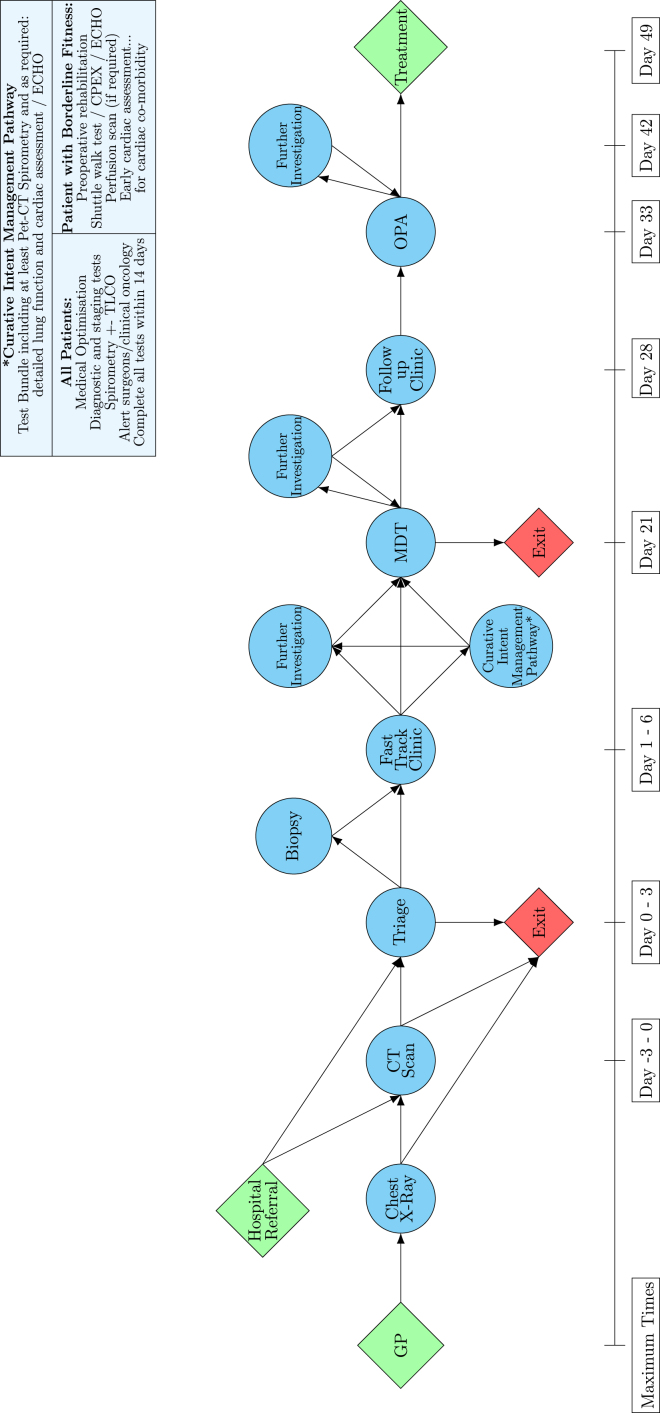
Simplified National Optimal Lung Cancer Pathway.

The pathway for cancer diagnosis starts at referral and ends at start of treatment, and contains many steps in between which detect the stage of the cancer. Within the UK there are different guidelines of how to conduct the cancer pathway, which are summarised in Table 2. In Wales, the National Optimal Lung Cancer Pathway (NOLCP) [99] is currently in place, and is currently in the process of being replaced by the Single Cancer Pathway [100]. For ease of understanding, we have converted the NOLCP to a simplified version just containing the activities and maximum time frames for completion (Appendix Fig. A.15).

Fig. A.15 (Appendix) shows that, a patient is rarely allowed to attend the same activity more than once. In fact, each activity was only recorded once in the dataset, putting a hard restriction on not allowing multiple attendances of an activity.

**Table 2:** UK and Ireland cancer pathway guidelines.

Country	Guideline	Provider
*England*	National Optimal Lung Cancer Pathway (NOLCP)	Cancer Research UK [99]
*Wales*	Single Cancer Pathway, National Optimal Pathway for Lung Cancer	Wales Cancer Network [100], NHS Wales [101]
*Scotland*	Management of lung cancer	Healthcare Improvement Scotland [102]
*Northern Ireland*	Lung Pathway	Northern Ireland Cancer Network [103]
*Ireland*	Lung Cancer Action Plan	Irish Cancer Society [104]

To adhere to this constraint all of the past performed activities would need to be considered when choosing the next activity to avoid duplication. As the memory-less property of Markov chains only allows the directly preceding activity to be considered, using Markov chains was not appropriate for our data. Therefore clustering was chosen as an appropriate method.

The data set used contains date stamps for each patient and each activity that was performed. To first extract the pathways from the data set, each activity is assigned a letter code, and then the activities are ordered by the date that they occurred, and joined together to form a string of letters.

For example, if a patient was first seen on 01/01/2019, then received a diagnosis on 02/01/2019 and then their case was discussed at a Multi-Disciplinary Team Meeting (MDT) on 03/01/2019, and these activities were assigned the letter codes A, B and C respectively, then the pathway for this patient would be ABC.

**Fig. 1:**
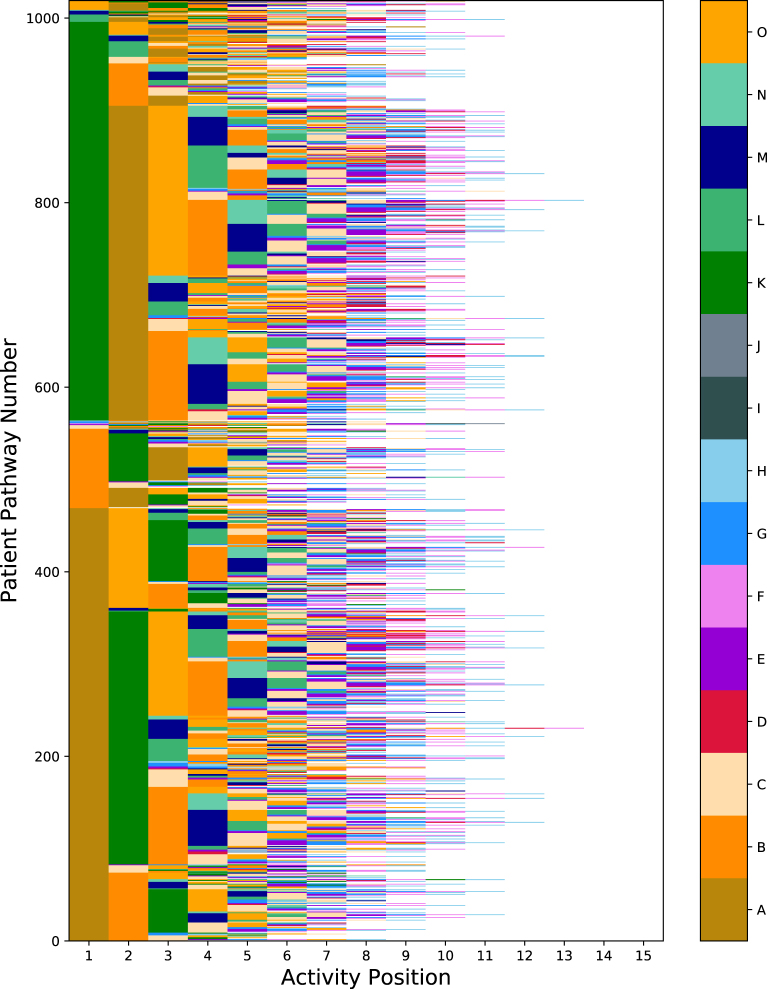
All patient pathways displayed as a heatmap.

To aid with visualisation of this, Fig. 1 shows a heatmap displaying the pathways, where the data has been ordered alphabetically. Along the x-axis is the position of the activity, and the y-axis is the number of patients, where each integer represents one patient. Furthermore, each activity code has been assigned a colour, and thus the heatmap represents the patient pathways as a line of various colours.

Fig. 1 shows that there is a large amount of variation in the position, number and sequence of the activities performed. This indicates that condensing this large variation into a simple clinical pathway to be used as guideline is a difficult task.

## 4 Description of metrics

There are many different possible metrics that can be used to compare two strings, given that the Python library textdistance [105] (a library to compare distance between two or more sequences) hosts over 30 algorithms for this purpose. Eight different metrics were considered to use as comparison and benchmarking for the modified algorithm, which cover edit distances, token based and sequence based distances. These eight metrics were chosen as they most appropriately fit the purpose, reflect the literature and show a variety of techniques.

### 4.1 Edit distances

Here there are five edit distances considered: Levenshtein, Damerau–Levenshtein, Jaro, Jaro–Winkler and Needleman–Wunsch.

***Levenshtein***

The Levenshtein distance was developed in 1965 for the use of correcting deletions, insertions and reversals of binary codes [106]. The general idea is to evaluate the distance between two strings as the number of single-character edits required to change one string into the other. There are many current uses for the Levenshtein distance, e.g. spell checkers, optimal character recognition correction systems and linguistic distance, to name a few.

**Fig. 2:**
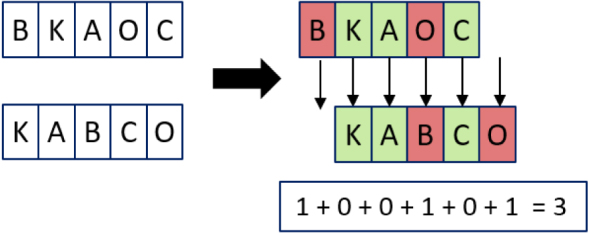
Example of the calculation for the Levenshtein distance.

The Levenshtein distance can easily be calculated by hand, by giving a penalty of one to each insertion, deletion or substitution, as demonstrated in Fig. 2.

**Fig. 3:**
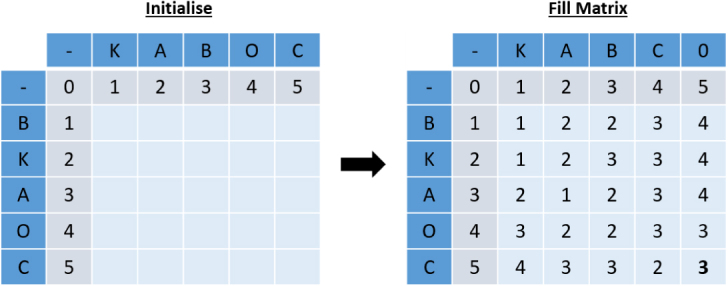
Example of dynamic programming using the Levenshtein distance.

The Levenshtein distance can be translated into a dynamic programming algorithm displayed in Algorithm 1. The dynamic programming matrix X for the example from Fig. 2 can be seen in Fig. 3.

**Figure:**
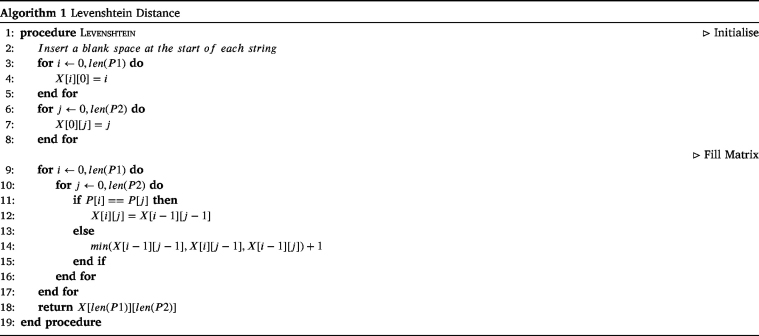

***Damerau–Levenshtein***

The Damerau–Levenshtein is an extension of the Levenshtein distance, where transpositions (swapping positions of adjacent letters) are also allowed [107].

**Fig. 4:**
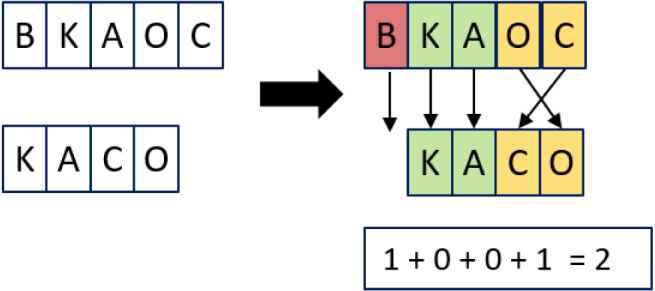
Example of the calculation for the Damerau–Levenshtein distance.

An example of the hand calculation for the Damerau–Levenshtein distance can be seen in Fig. 4. Again, this can also be performed using dynamic programming.

***Jaro***

The Jaro similarity was first developed for the purpose of record linkage [108], [109]. The formula considers four variables: the length of both strings (a,b), the number of matching characters (m) within a tolerance (T), and the number of transpositions within those matching characters (t). The formula for Jaro similarity is as follows:

(1) $$sim_{jaro}=\left\{\begin{array}{cc} 0,\; & \text{if m}=0\\ \frac{1}{3}\left(\frac{m}{a}+\frac{m}{b}+\frac{m-t}{m}\right),\; & \text{otherwise} \end{array}\right)$$

where the tolerance (T) for m is calculated by

$$\left[\frac{\max\left(a,b\right)}{2}\right]-1,$$

and only the integer-part is used. For further clarity, two characters are only considered matching if they are within T places of each other.

This will produce a value between 0 and 1, where 1 indicates that the strings are identical, and therefore a larger value is desired.

To calculate the distance instead of similarity, the metric needs to be adjusted by performing $1-sim_{jaro}$.

**Fig. 5:**
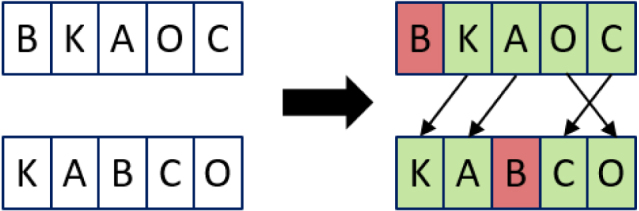
Example of the calculation for the Jaro distance. (For interpretation of the references to colour in this figure legend, the reader is referred to the web version of this article.)

For example, in Fig. 5 there are 4 matches within the tolerance of 1 (see below), shown in green, however C and O need to be transposed.

The calculations for the example in Fig. 5 are: a = 5, b = 5, T = 5/2 − 1 = 1, m = 4, t = 1

(2) $$sim_{jaro}=\frac{1}{3}\left(\frac{4}{5}+\frac{4}{5}+\frac{4-1}{4}\right)=0.78\overset{˙}{3}$$

(3) $$1-sim_{jaro}=0.21\overset{˙}{6}$$

***Jaro–Winkler***

The Jaro–Winkler distance is an extension of the Jaro distance [110] through the following formula:

(4) $$sim_{winkler}=sim_{jaro}+\left(l*p\left(1-sim_{jaro}\right)\right)$$

where l is in number of common prefix before the first non-match, up to a maximum of 4, and p is a scaling factor which should not exceed 0.25. Typically p is chosen to be 0.1. Again, to calculate the distance instead of similarity, the metric needs to be adjusted by performing $1-sim_{winkler}$.

**Fig. 6:**
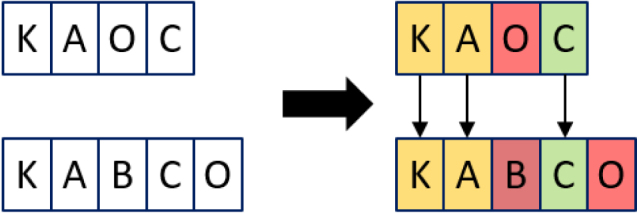
Example of the calculation for the Jaro–Winkler distance.

Applying this calculation to the example, as l is 0 (because the first position is a non-match), we would get the same result as the Jaro distance (0.2166666667). Therefore, the example is changed slightly as shown in Fig. 6.

Then first calculating the Jaro distance to allow for calculating the Jaro–Winkler distance is as follows: a = 4, b = 5, T = 5/2 − 1 = 1, m = 3, t = 0

(5) $$sim_{jaro}=\frac{1}{3}\left(\frac{3}{4}+\frac{3}{5}+\frac{3-0}{3}\right)=0.78\overset{˙}{3}$$

(6) $$1-sim_{jaro}=0.21\overset{˙}{6}7$$

(7) $$sim_{winkler}=0.78\overset{˙}{3}+\left(2*0.1\left(0.21\overset{˙}{6}\right)\right)$$

(8) $$=0.82\overset{˙}{6}$$

(9) $$1-sim_{winkler}=1-0.82\overset{˙}{6}=0.17\overset{˙}{3}$$

***Needleman–Wunsch***

The Needleman–Wunsch algorithm was first used in bio-informatics to align protein or nucleotide sequences, and makes use of dynamic programming [111]. It may also be referred to as the optimal matching algorithm or the global alignment technique.

**Fig. 7:**
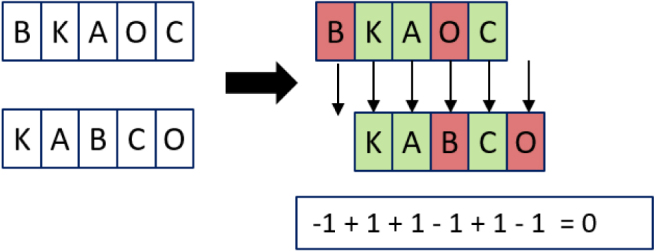
Example of the calculation for the Needleman–Wunsch distance.

This is a generalised variant of the Levenshtein distance, where values for match, swap and gap are chosen by the user. The most common values chosen for these variables are: Match (m) = 1, Swap (s) = −1 and Gap (g) = −1. Again, this can easily be checked by hand, as shown in Fig. 7.

The Needleman–Wunsch algorithm also makes use of dynamic programming to computationally calculate the distance. The pseudo-code for which can be seen in Algorithm 2.

**Figure:**
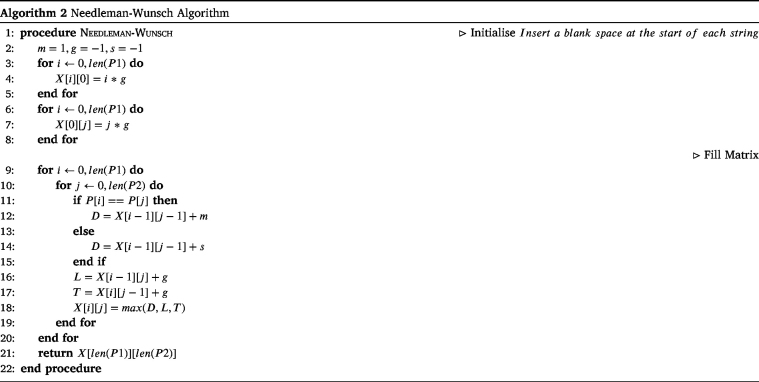

**Fig. 8:**
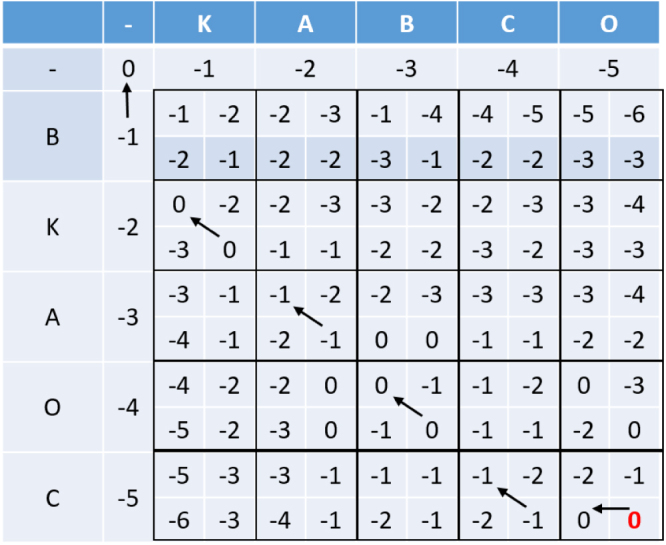
Example of the Needleman–Wunsch algorithm.

An example of the matrix produced using the Needleman–Wunsch dynamic programming algorithm can be seen in Fig. 8.

Once the matrix such as that in Fig. 8 has been produced, we can perform traceback to find the alignment. This means, starting at the bottom-right corner of the matrix, and working back through the matrix to the top-left 0, and noting the direction that the value came from. This is highlighted in Fig. 8 by the black arrows.

A diagonal arrow indicates an alignment, an arrow to the left indicates that the character in the left string is aligned with a gap, and an arrow straight up indicates that the character in the top string is aligned with a gap.

The textdistance library [105] does not easily allow for alternative values of m, s and g to be used.

### 4.2 Token based distances

Here are discussed two token based distances, Jaccard and Cosine respectively. In this context token means a partition of the string, and in both of these distances this relates to n-grams. Furthermore, an n-gram is defined as a continuous sequence of n items.

***Jaccard distance***

The Jaccard distances [112] is calculated using the following equation.

(10) $$\frac{\left|A\cup B\right|-\left|A\cap B\right|}{\left|A\cup B\right|}$$

**Fig. 9:**
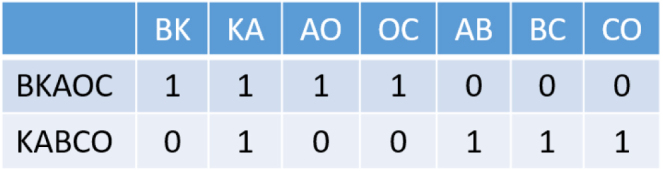
Example of bi-gram for Jaccard distance.

An example of n-grams, where n = 2 (bi-gram), for the two strings BKAOC and KABCO, as required for the Jaccard distance can be seen in Fig. 9. Applying the formula to this example yields:

(11) $$\frac{6-1}{6}=\frac{5}{6}=0.8\overset{˙}{3}$$

***Cosine distance***

The Cosine distance is typically used to compare the number of similar words in a document and also in data mining to measure cohesions in clusters [113].

Firstly, calculating the Cosine similarity of both n-grams, using the following equation:

(12) $$\frac{\overset{n}{\underset{i=1}{\sum }}A_{i}B_{i}}{\sqrt{\overset{n}{\underset{i=1}{\sum }}A_{i}^{2}}\sqrt{\overset{n}{\underset{i=1}{\sum }}B_{i}^{2}}}$$

Then, as previously, 1 minus the similarity needs to be performed to obtain the distance. Applying this calculation to the example with previously calculated the n-grams in Fig. 9. This results in a cosine distance of 1−0.25=0.75.

$$\overset{n}{\underset{i=1}{\sum }}A_{i}B_{i}=\left(1*0\right)+\left(1*1\right)+\left(1*0\right)+\left(1*0\right)+\;\left(0*1\right)+\left(0*1\right)+\left(0*1\right)=1\overset{n}{\underset{i=1}{\sum }}A_{i}^{2}=1^{2}+1^{2}+1^{2}+1^{2}+0^{2}+0^{2}+0^{2}=4\overset{n}{\underset{i=1}{\sum }}B_{i}^{2}=0^{2}+1^{2}+0^{2}+0^{2}+1^{2}+1^{2}+1^{2}=4$$

$$\frac{1}{\sqrt{4}\sqrt{4}}=0.25$$

### 4.3 Sequence based distances

***Longest common subsequence***

**Fig. 10:**
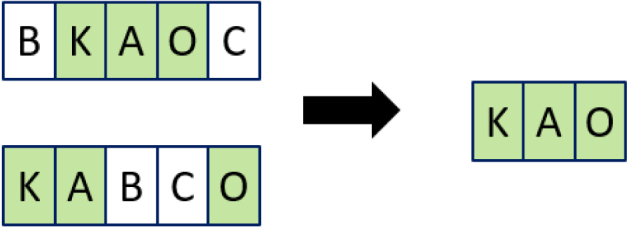
Example of longest common subsequence.

The longest common subsequence (LCS) refers to the longest subsequence common to both sequences, where the subsequences do not have to occupy consecutive positions, but do have to be in sequence. Fig. 10 displays that the LCS for our example is 3.

To consider LCS as a distance, we need to consider what remains when you remove the LCS. In Fig. 10 this would be what remains in white, and therefore would give a LCS distance of 2.

It has been shown that this is an NP-hard problem [114], and as such dynamic programming has been utilised to allow for computation. The pseudo-code for the dynamic programming of the LCS can be seen in Algorithm 3.

**Fig. 11:**
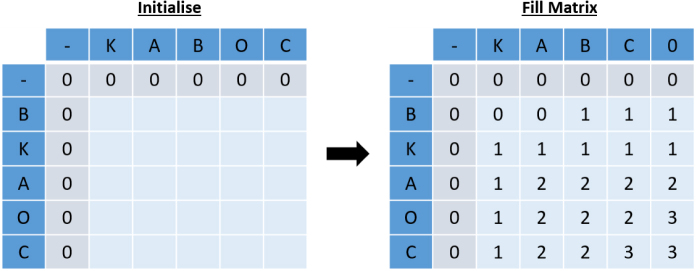
Example of longest common subsequence.

Fig. 11 illustrates the dynamic programming calculation for the example in Fig. 10.

**Figure:**
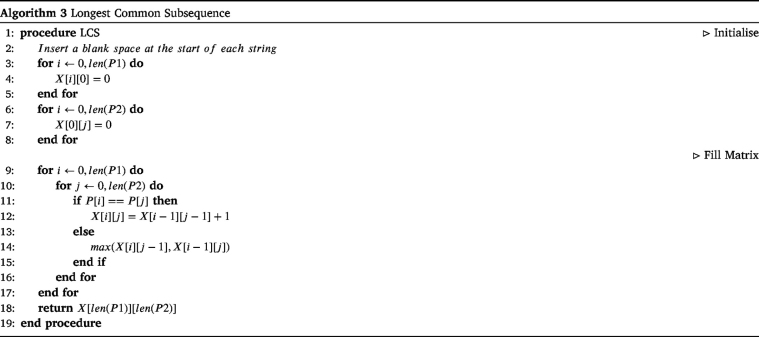

### 4.4 Properties of metric

When selecting an appropriate distance metric, it is important to consider which properties are important when calculating similarity. There are three key properties that can be considered with string metrics, namely length, sequence and position.

*Length*: It is apparent that considering strings of differing length is a common occurrence in process data, in particular with medical diagnosis, as it is a process of discovery and one that may need different activities based on the results of a previous one. Therefore, the algorithm needs to consider the differing length of two strings.

*Sequence*: The sequence in which activities occur is important and must be considered, especially when considering the previous statement that the results of one activity may change the course of the pathway.

*Position*: The position that the activity, and the sequence of activities, occurs within the pathway is vitally important to consider when developing an algorithm for process data.

All of these properties are considered in varying degrees in each of the eight metrics considered in the previous section. For example, *length* is evidently considered in the Jaro calculation, as it is a main variable in the formula, whereas in the Levenshtein distance *length* is indirectly considered via the upper and lower bounds for the possible values (upper bound = length of the longer string, lower bound = the difference between the lengths of the strings). Furthermore, *sequence* is evidently considered in LCS, as it is in the name, whereas *sequence* is considered in an alternative way in the Jaccard distance through the use of n-grams.

This shows that string distance metrics do possess the correct qualities to be applied to process data.

The string distances are currently underperforming when considering small differences between strings. The addition of an extra letter will be considered, but it does not make a difference what letter it is or what it represents. This leads to many string comparisons resulting in the same value (as seen in Appendix Fig. A.17, Fig. A.18). It is theorised that this will lead to poor cluster distinction and adding some uniqueness will improve upon this.

In attempt to address this it was evident that complete uniqueness was difficult to achieve as it violated some fundamental basic relationships (such as symmetry). However, adding more uniqueness than is currently displayed in the distance metrics was successful.

Addressing the previous property, allowed for the ability to include some meaning to the strings. As discussed previously, there is no consideration in the metrics for *which* letter has been added and what that might represent. This is likely due to the origins of the metrics typically being for spell checkers etc. where there is no need to consider this. However, in terms of process data, it can cause quite a difference when considering the addition of letter A or letter B depending on what activities they represent.

In summary, we aim to modify the Needleman–Wunsch algorithm to allow for more uniqueness in the values to achieve better clustering results, through the addition of context provided by experts. The process for this is explained in more detail in context in Section 5.

## 5 Modified Needleman–Wunsch algorithm

This section discusses the development of the Modified Needleman–Wunsch algorithm to achieve adding uniqueness and context to the comparisons.

The Needleman–Wunsch metric was chosen as the base for this modification, as it had the greatest potential to modify the calculation in a meaningful way. As the intention for this modified metric was to be applied to clustering process data, three fundamental properties need to be preserved: (1) a point to itself receives a score of 0, (2) symmetry must hold and (3) a smaller value is indicative of a closer match. This will be addressed in the discussion concerning penalty values.

### 5.1 Variables

The first modification considered is the idea that not all activities should be allowed to swap with each other. This is because, considering the pathway from a resource planning perspective and the interaction between multiple care centres, allowing all activities to swap could lead to very different pathways being considered similar. For example, allowing an X-ray under primary care supervision, is very different from an MDT meeting consisting of multiple personnel from the secondary and tertiary centres, from a resource perspective.

To allow for this, a no-swap variable (ns) needs to be defined. Furthermore, the algorithm needs to be able to decipher which activities are allowed to swap with each other. This leads to the introduction of groups of activities, where essentially, if activities are in the same group then they are allowed to swap.

### 5.2 Groupings

The experts will be asked to group activities that happen at similar points in the pathway into the same group. It should be explained that the purpose of these groups is that if two patients performed different activities at the same point in their pathway, but these activities are in the same group, then they would be seen as more similar to each other than if the activities were in different group. An example of the groupings used for the case study are provided in Table 3.

This permits greater meaning to be given to the pathways, however this does not lead to the values being more unique. This is addressed by using weightings and is discussed in the next section.

**Table 3:** Grouping assignments for each activity.

Group	Activities
0	A,B,C,O
1	D
2	E,F
3	G,H
4	I,J
5	K,L,N
6	M

### 5.3 Weightings

The inclusion of weightings into the algorithm increased the complexity, and as such now becomes more difficult to calculate by hand.

We first discuss how to assign the weightings to the activities, and then follow with combining these into the algorithm.

Assume that domain experts (e.g. consultants in cancer services) are asked to rank the activities from most to least important (0 to N-1, where N is the number of activities). This can be thought of as, the activity that occurs most often is seen as most important, and thus ranked 0, and those activities that are more rarely occurring should be ranked as lesser important. From these rankings, they will then be converted into weightings where the least important activity will be assigned a weight of 1, and each activity will receive an incremental addition of 1/(N-1). This subsequently gives the most important activity a weight of 2.

For example, Table 4 shows the rankings and resulting weightings (rounded to 3 d.p.) that were applied to the case study activities.

**Table 4:** Ranking and weighting results for each activity.

Activity	Rank	Weighting
A	2	1.857
B	0	2.0
C	1	1.929
D	12	1.143
E	10	1.286
F	9	1.357
G	7	1.5
H	6	1.571
I	13	1.071
J	14	1.0
K	3	1.786
L	5	1.643
M	8	1.429
N	11	1.214
O	4	1.714

### 5.4 Equations

As we have now defined both the groupings and weightings, we can combine these into the algorithm. We will first methodically work through the equations, including explanations, and then provide the pseudo-code.

Firstly, the match equation is as follows:

(13) $$D=X\left[i-1\right]\left[j-1\right]*\left(m+\frac{1}{X\left[i-1\right]\left[j-1\right]+w_{i}}\right)$$

The match equation had to be modified using multiplication of the m parameter, to allow the initial 0 to propagate through. This is the main element that allows for a point to itself to be 0 (as required by the fundamental properties of metrics introduced in the beginning of Section 5).

The inclusion of the previous matrix value (X[i-1][j-1]) is required in the denominator to control the magnitude, and ensure that the penalty value for a match will not exceed 1.

Furthermore, as a match is a positive event, we needed to ensure that in this case, a more important activity has a smaller impact than a lesser important activity. This is the reason for the 1 over weight.

Moving on to the swap equation:

(14) $$D=X\left[i-1\right]\left[j-1\right]+s+abs\left(w_{i}-w_{j}\right)$$

This is more intuitive, as the modification is the addition of the absolute difference of the two weightings. This results in activities that are allowed to swap, but are ranked further apart will have a larger value than those that are ranked closer.

Now considering the no-swap equation:

(15) $$D=X\left[i-1\right]\left[j-1\right]+ns+\left(w_{i}+w_{j}\right)$$

This ensures that the no swap value is large enough to never get chosen in the matrix.

The gap equations are only slightly modified through the addition of the corresponding weighting of that direction:

(16) $$L=X\left[i-1\right]\left[j\right]+g+w_{i}$$

(17) $$T=X\left[i\right]\left[j-1\right]+g+w_{j}$$

The final modification from the Needleman–Wunsch algorithm is that now we select the minimum of D, L, T opposed to the maximum. Algorithm 4 displays the pseudo-code for the modified Needleman–Wunsch algorithm.

**Figure:**
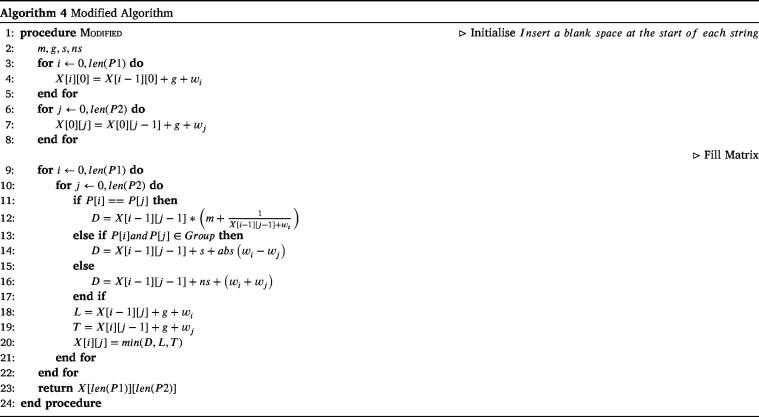

### 5.5 Penalty values

In the literature surrounding the Needleman–Wunsch algorithm, it is often discussed that the user can specify the values for the match, swap and gap penalty, however there are no guidelines surrounding these.

We developed the following equations as guidelines, to ensure that the preference of, match < swap < gap < no-swap, holds when choosing values for the variables.

1<g

1<s≤g

ns=2g+1

m=1

For further clarification, m must be set to 1 as the match equation considers a multiplication, and otherwise the factor is not consistently less than 1 (more clarification below). Moreover, it is unnecessary for ns to be larger than 2g + 1, as this is sufficient to consistently force gaps when a no swap is necessary.

As a result, the smallest possible penalty values are: m=1, g=2, s=2, ns=5.

As with the standard Needleman–Wunsch algorithm, changes to the penalty values will result in different distances calculated, which will propagate through to the clustering. Advice to the user when selecting the values of s and g in particular, is to select values with a larger difference between s and g to ensure a more distinct separation of these two actions.

**Fig. 12:**
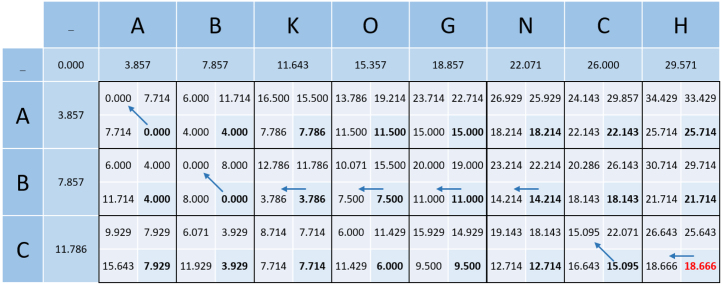
Example of modified dynamic programming algorithm.

**Fig. 13:**
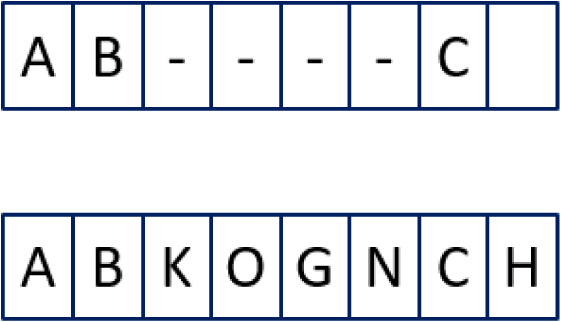
Example of modified traceback.

**Fig. 14:**
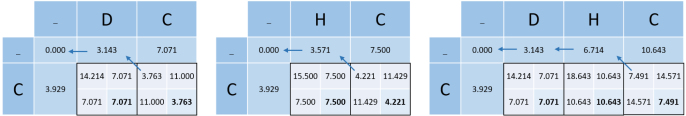
Example of feature five.

### 5.6 Example

Fig. 12 calculates the modified Needleman–Wunsch distance between the two pathways ABKOGNCH and ABC, using the values m = 1, g = 2, s = 2 and ns = 5, with the groupings and weightings from Table 3, Table 4 respectively.

Fig. 13 shows the resulting alignment from following the traceback.

Consider that intuitively it should always be better to take a swap over a gap. However, looking at the interaction between B and O, it can be seen that this is not the case, as the value from the gap is smaller than that of the allowed swap. At first glance, this may seem incorrect, until further inspection when it is clear that this is necessary to allow the alignment of B with itself two steps later.

This demonstrates the intelligence of the algorithm, and the consideration for the string as a whole during traceback.

### 5.7 Features

The modified algorithm allows for many features to be considered, which are as follows:

1. Point to itself is 0
2. The distance score for the string is 0 until the first non-match (similar to the common prefix idea in the Jaro–Winkler distance)
3. Distances between two pathways are commutative
4. Matches between higher importance activities produce a smaller distance
5. A match earlier in the string will result in a smaller value than that appearing later
6. Gaps with higher importance activities are larger value than that of lower importance
7. Swaps of activities that are closer in terms of rankings will produce a smaller value

**Fig. A.16:**
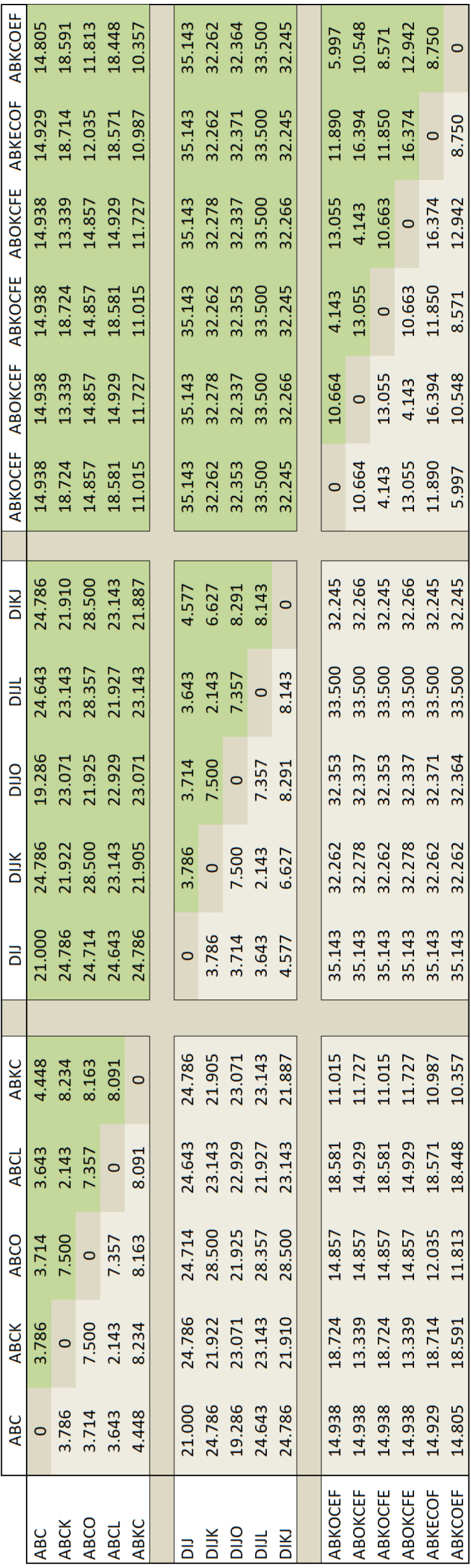
Modified Needleman–Wunsch Distance Matrix for Sample 2.

Fig. A.16 (Appendix) displays all the features described above for Sample 2 (explained below) using penalty values m = 1, g = 2, s = 2, ns = 5.

To add commentary to Fig. A.16 (Appendix), feature 1 is displayed along the diagonal of the matrix, and feature 3 (commutativity) is displayed, and thus one can ignore the bottom diagonal of the matrix, and just examine the top diagonal.

Feature 2 can be confirmed by matrix locations (1,2), (1,3) and (1,4), as the value corresponds to g with the addition of the weight for the additional letter as displayed in Table 4. These three values also confirm feature 6.

Features, 4 and 7, are displayed amongst Fig. A.16 (Appendix), but can easily be checked manually by combining the weightings in Table 4 with the equations for the match and swap (Eqs. (13), (14)) respectively.

Feature 5 is the most complex and a by-product of feature 1. This feature arises due to the match penalty calculation being a factor or the previous value (as previously discussed in the context of Eq. (13)). This feature can be seen in matrix locations (1,2) compared to (1,5), where (1,2) is smaller than (1,5) as the match of C happens earlier in (1,2) than in (1,5). To further display this feature, consider the string C compared with the following three string: (1) DC, (2) HC, and (3) DHC. Fig. 14 shows the full calculation matrix of each of the three scenarios. If we calculate the impact of matching C in each scenario by observing the difference between the two values (indicated by the diagonal arrow in Fig. 14), as follows:

(18) (1)3.763−3.143=0.62(2)4.221−3.571=0.65(3)7.491−6.714=0.777

Eq. (18) shows that the penalty for matching C is different in all three scenarios. Simplified, if the previous value is larger then the effect of matching C is also larger. Hence, the later a match appears in the string, the larger the value.

In conclusion, the modified Needleman–Wunsch algorithm does produce a more specific value for distance, considering length, position, and sequence, whilst also considering the weightings and groupings of the activities.

## 6 Case studies

Our research applies the eight previously discussed metrics and the modified algorithm to two small samples and the full case study dataset. These samples are very basic to allow the reader to closely examine the intricate differences that appear due to the inclusion of the weighting and rankings. Furthermore, sample 1 and sample 2 are easily assigned to two and three groups respectively, to display that the obvious solution is found in a simple example, and to provide the reader with confidence when applying this to more complex data. Although these samples are artificially constructed, they reflect the small differences between strings seen in practice.

Sample 1 consists of 10 pathways: ABC, ABCK, ABCL, ABCO, ABKC, DIJ, DIJK, DIJL, DIJO, and DIKJ. These were chosen as A,B,C and D,I,J are the highest and lowest ranked activities respectively.

Sample 2 consists of 16 pathways, the same 10 as in sample 1, plus a further six which display the complexity of allowed swaps between slight differences within the pathway. These are: ‘ABKOCEF’, ‘ABOKCEF’, ‘ABKOCFE’, ‘ABOKCFE’, ‘ABKECOF’, ‘ABKCOEF’.

Two examples of the modification are included in the analysis using penalty values g = 2, s = 2, ns = 5 and g = 9, s = 2, ns = 19, which will be referred to as MNW_1225 and MNW_19219 respectively.

The analysis for the two samples is as follows: Firstly, the distances between all the points are calculated using the ten previously discussed metrics, and then plotted to demonstrated how the modified algorithm allows for more separation in the data. Secondly, the k-medoids clustering is run for k = [2,8], where the use of the silhouette scores both confirms point one and displays that the modified algorithm outperforms most of the other metrics. The findings are displayed in a table, which contains the results for k = 2 and then the best performing k (if k = 2 was best, then the second best is displayed), which includes the number of iterations.

The following python libraries were used: textdistance [105] was used for calculations of the eight other distance metrics, pyclustering [115] was used for the k-medoids clustering and scikit-learn was used for the calculation of the silhouette score [116].

### 6.1 Sample 1: 10 pathways

**Fig. A.17:**
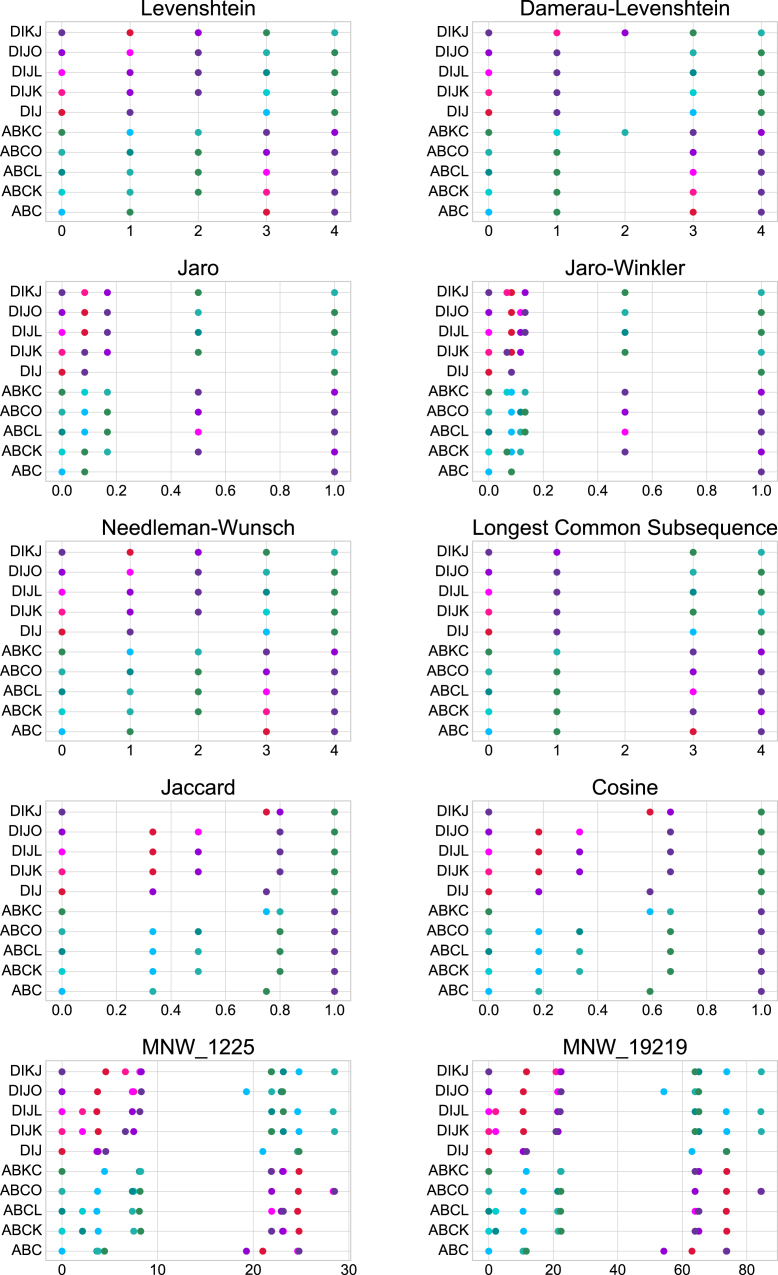
Comparison of the Ten Metrics Applied to Sample 1. (For interpretation of the references to colour in this figure legend, the reader is referred to the web version of this article.)

Fig. A.17 (Appendix) displays a comparison of the distances between the pathways in sample 1 for each of the eight measures discussed in Section 4 and the two examples of the modified algorithm (MNW_1225 and MNW_19219).

To aid understanding of Fig. A.17 (Appendix), firstly the distance from each point to itself is 0, and therefore the colour of the dot at x = 0 for each pathway on y is the colour that represents that pathway e.g. pathway DIJ is represented by the red dot. Furthermore, all pathways beginning with A are from the blue colour pallet, and those beginning with D are from the red colour pallet.

The y-axis displays the pathway which all others are being compared to and the x-axis displays the distance from that pathway. For example, in the top left graph considering the Levenshtein distance, the distance from ABC (light blue) to ABKC (dark green) is 1.

In all eight of these graphs in Fig. A.17 (Appendix), if you split the graph horizontally between ABKC and DIJ, and overlaid the two halves, you can see that the distances are exactly the same, and reflects the lack of uniqueness. There is also little separation between the blue and red groups, with the exception of the Jaro and Jaro–Winkler graphs, where this is more clear.

Now considering the bottom two graphs in Fig. A.17 (Appendix), which display the modified algorithm (penalty values g = 2, s = 2 and ns = 5 on the left and g = 9 s = 2 and ns = 19 on the right). It can clearly be seen that this algorithm allows for more uniqueness and greater separation between the colour groups, as desired.

To confirm that this is reflected in the clustering, k-medoids clustering was performed for all ten metrics, the results for which are displayed in Table 5. The initial centroids were chosen as 0: ‘ABC’ and 5: ‘DIJ’. It is expected that the clustering algorithm should keep ‘ABC’ and ‘DIJ’ as the centroids.

Table 5 displays the expected results, with the only measures that surpass the modified Needleman–Wunsch in silhouette score is the Jaro and Jaro–Winkler.

**Table 5:** Clustering of Sample 1, for all ten distances.

Name	Centroids	Number per cluster	Silhouette score
Levenshtein	0, 5	5, 5	0.65789
Damerau–Levenshtein	0, 5	5, 5	0.70614
Jaro	0, 5	5, 5	0.85602
Jaro–Winkler	0, 5	5, 5	0.88333
Needleman–Wunsch	0, 5	5, 5	0.65789
Jaccard	0, 5	5, 5	0.43500
Cosine	0, 5	5, 5	0.58577
LCS	0, 5	5, 5	0.73099

MNW_1225	0, 5	5, 5	0.76128
MNW_19219	0, 5	5, 5	0.77464

### 6.2 Sample 2: 16 pathways

**Fig. A.18:**
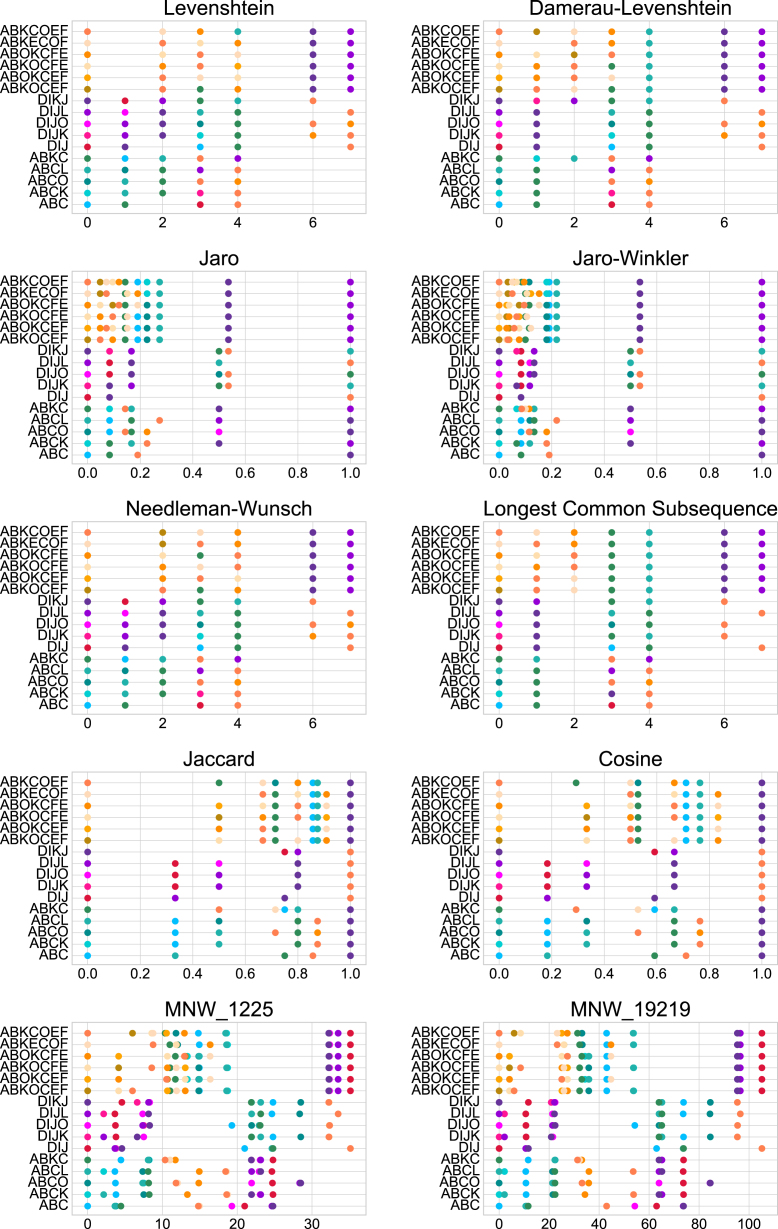
Comparison of the Ten Metrics Applied to Sample 2. (For interpretation of the references to colour in this figure legend, the reader is referred to the web version of this article.)

Similarly to the previous subsection, Fig. A.18 (Appendix) displays a comparison of the distances between the pathways in sample 2 for each of the eight measures discussed in Section 4 and the two examples of the modified algorithm (MNW_1225 and MNW_19219).

In this sample, it is logical to assume that three clusters would be appropriate, the same two as in sample 1 and a further one containing the extra six pathways. Therefore Fig. A.18 (Appendix) should be examined for the appearance of three distinct groups.

This is actually not as clear cut as it was with sample 1 (in relation to two groups). In the majority of the metrics, it is difficult to find the clear groups one is expecting (one group of red, one group of blue and another of yellow). Again the distinction is more clear in the modified algorithm, especially with the penalty values g = 9, s = 2 and ns = 19 (as previously stated). This further confirms that the modified algorithm allows for better distinction between pathways.

To confirm if this is reflected in the clustering, the same analysis was run as that described for sample 1, where the initial centroids were chosen as 0: ‘ABC’, 5: ‘DIJ’ and 10: ‘ABKOCEF’, and for k = [2,3]. It is expected that the clustering algorithm should keep the same centroids, and that three clusters would be chosen.

Table 6 confirms that the modified algorithm performs equally well as the other metrics, and selects the expected centroids, which is not the case with some of the other metrics.

**Table 6:** Clustering of Sample 2, for all ten distances.

Name	Centroids k = 2	Number per cluster k = 2	Silhouette score k = 2	Centroids k = 3	Number per cluster k = 3	Silhouette score k = 3
Levenshtein	4, 5	11, 5	0.51433	0, 5, 10	6, 5, 5	0.45234
Damerau–Levenshtein	4, 5	11, 5	0.54800	0, 5, 10	5, 5, 6	0.62230
Jaro	5, 10	5, 11	0.80971	0, 5, 10	5, 5, 6	0.58120
Jaro–Winkler	4, 5	11, 5	0.84600	0, 5, 10	5, 5, 6	0.60252
Needleman–Wunsch	4, 5	11, 5	0.50676	0, 5, 10	6, 5, 5	0.43148
Jaccard	0, 5	11, 5	0.30516	0, 4, 5	4, 7, 5	0.32543
Cosine	0, 5	11, 5	0.44807	0, 4, 5	4, 7, 5	0.43025
LCS	4, 5	11, 5	0.56353	0, 5, 10	5, 5, 6	0.67356

MNW_1225	4, 5	11, 5	0.64700	0, 5, 10	5, 5, 6	0.53059
MNW_19219	4, 5	11, 5	0.67874	0, 5, 10	5, 5, 6	0.59195

**Table 7:** Results of full data clustering for k = 2.

Name	Iter	Medoids	Pathways per cluster	Score
Levenshtein	3	KAOBC, AKBMCEGFH	663, 356	0.15604
Damerau–Levenshtein	2	KAOBCD, AKBMCEGFH	676, 343	0.17549
Jaro	3	KAOBLCD, AKOBMCEGFH	409, 610	0.18343
Jaro–Winkler	3	KAOBCD, AKOBMCEGFH	445, 574	0.17542
Needleman–Wunsch	2	AOBC, AOBCEGFH	727, 292	0.16743
Jaccard	2	KAOBNLCGH, KAOBMCEGFH	650, 369	0.04297
Cosine^a^	2	KAOBNLCGDH, KAOBMCEFGH	649, 369	0.06854
LCS	2	KAOBCD, KAOBCEGFH	510, 509	0.24305

MNW_1225	2	KABC, AOBCEGFH	715, 304	0.14303
MNW_19219	2	AOBC, AKOBCEGFH	676, 343	0.17976

a For cosine, the pathway consisting of just activity B had to be removed, as it caused division by 0.

**Table 8:** Results of full data clustering for best k (exclusing k = 2).

Name	k	Iter	Medoids	Pathways per cluster	Score
Levenshtein	3	4	KAOBC, AKBMCEGFH, ABCO	541, 348, 130	0.06964
Damerau–Levenshtein	3	4	KAOBCD, AKBMCEGFH, ABKOC	519, 333, 167	0.09724
Jaro	3	3	KAOBLCD, KAOBMCEGFH, ABKOC	315, 503, 201	0.16252
Jaro–Winkler	3	3	KAOBLCD, KAOBMCEGFH, ABKOC	308, 487, 224	0.16254
Needleman–Wunsch	3	2	AOBC, AOBCEGFH, ABCO	582, 279, 158	0.06689
Jaccard	7	3	KAOBNLC, KAOBMCEGFH, KAOBC, AKOBNC, KABNCOEF, AOKBMC, BKAOCGH	137, 229, 117, 194, 84, 113, 145	0.05322
Cosine^a^	7	4	KANOMBCEFD, KAOBMCEFGH, ABC, AKOBC, KABMCO, AOKBC, BKAOCEGFH	172, 219, 45, 207, 122, 89, 164	0.08812
LCS	3	3	KAOBCD, KAOBCEGFH, ABKC	408, 509, 102	0.14132

MNW_1225	3	4	KAOBC, AOBCEGFH, AKBC	384, 199, 436	0.13354
MNW_19219	3	4	AKOBC, AOKBCEGFH, KAOBC	403, 229, 387	0.14860

a For cosine, the pathway consisting of just activity B had to be removed, as it caused division by 0.

It was expected that three clusters should be chosen, however, examining the silhouette scores it appears that in most cases the score for k = 2 is closer to 1 than in k = 3, suggesting that two clusters is better. This indicates that possibly the silhouette score is not the most appropriate measure to use, and care is needed when selecting the appropriate number of clusters.

In conclusion both samples display that the modified algorithm does enhance the differences between strings based on user specific characteristics, and performs equally well, if not better, than the currently used metrics.

### 6.3 Full data

This section applies the eight measures discussed in Section 4 and the two examples of the modified algorithm (MNW_1225 and MNW_19219) to the full data set which was discussed in Section 3. As a recap, there are 2350 patients and 1019 different pathways considering the 15 activities. We have applied k-medoids clustering to the data, considering values of k = [2,8] and initial centroids as [0,1,2,3,4,5,6,7].

Table 7 shows the results for k = 2 and Table 8 for the (next) best value of k (in terms of silhouette score). Both tables also include the medoids that were chosen and the number of pathways assigned to each of those cluster medoids.

The run time for each distance matrix was under 10 min, where the modified Needleman–Wunsch algorithm performed within the range of the other metrics.

Both Table 7, Table 8 shows that the silhouette scores for all 10 measure are quite poor. However, the silhouette score for the Needleman–Wunsch modification, with both sets of penalty values, is on par with the other metrics for k = 2 (with the exception of LCS), and surpass most of the other measure, with the exception of the jaro metrics when considering the second best value for k. This shows that for a full dataset, the modification performs equally as well, if not better, than the frequently used metrics when considering the silhouette score.

Furthermore, the metrics as a whole do not come to a consensus on a solution for the clustering as each of the metrics produce different results when considering the centroids selected and the number of pathways assigned to each cluster. Even when the same medoids are selected, the number of pathways assigned to those medoids clusters are not the same. This confirms that careful consideration is needed when selecting the distance metric, and what differences are to be highlighted.

## 7 Conclusions

A recent review of the literature [5] highlighted that clustering is a popular method for pathway discovery, however the distance metrics that apply to string data are lacking in uniqueness and do not hold any context. The review [5] also highlighted the lack of techniques that consider both information gathered from data and experts together, when developing a clinical pathway.

As a result, this paper discusses the development of a new distance metric, modified from the Needleman–Wunsch dynamic programming algorithm, that is specifically designed for clustering, and allows for expert interaction through the use of groupings and rankings of activities.

The modified metric was compared against eight other popular metrics, where it performed equally well, if not better, when used with k-medoids clustering. This comparison further highlight that each of the metrics produce different results and as such, confirms the hypothesis that careful consideration is needed when selecting a string metric.

Care needs to be taken when selecting the penalty values along with the rankings and groupings as the values selected here will change the results produced by the clustering. Further work could be considered here to aid the user in how to most effectively select the values here.

This method can support clinical pathway redesign or optimisation by initially providing a more time efficient process for mapping clinical pathways through combining both data and expert knowledge. As a result of combining both data and expert knowledge the clusters should be more clinically relevant using the modified Needleman–Wunsch metric due to the rankings and groupings feature.

From a clinical perspective, the resulting clusters enable deeper examination of the activity interactions which can help to highlight patterns that were previously undetectable when looking at the data as a whole. This can support decision makers in the pathway redesign process which could lead to reducing delays to diagnosis and improved outcomes. This can also allow decision makers to prospectively consider the capacity required at activities due to a awareness of preceding activity demand.

To further facilitate the use of this method, the modified algorithm (including the rankings and groupings feature) have been built into a decision support tool, Sim.Pro.Flow, which is available open access on Github [117]. Sim.Pro.Flow supports further exploration of the resulting clusters through allowing visualisation of the pathways as a network and allowing the pathways to be explored through a discrete event simulation.

Overall, the modified metric paves the way to adding more context to string distances, and bridges the gap between data and expert interaction.

**Further work**

The following areas have been identified as further work:

• Smart selection of penalty values: Machine learning techniques could be utilised to select penalty values which highlight various relationships as appropriate.
• Modify the Jaro distance metric [108], [109] using the same idea, as it produces good silhouette scores.
• Consider a final adjustment to the modified value to account for the total number of letters that appear in both strings i.e. divide final value by the number of letters appearing in both.
• Further sensitivity analysis to aid guidance in selecting penalty values, rankings and groups.
• Investigation of the impact of allowing groupings of singular activities, and how this could be used effectively.

## CRediT authorship contribution statement

**Emma Aspland:** Methodology, Formal analysis, Software, Writing - original draft. **Paul R. Harper:** Conceptualisation, Supervision, Writing - review & editing. **Daniel Gartner:** Conceptualisation, Supervision, Writing - review & editing. **Philip Webb:** Data curation, Supervision, Writing - review & editing. **Peter Barrett-Lee:** Supervision, Writing - review & editing.

## Declaration of Competing Interest

The authors declare that they have no known competing financial interests or personal relationships that could have appeared to influence the work reported in this paper.
